# Establishing Evidence for the Painful Intercourse Self-Efficacy Scale—Interstitial Cystitis

**DOI:** 10.1089/whr.2024.0152

**Published:** 2025-03-27

**Authors:** A. Grace Kelly, Susanna L. Sutherland, Elizabeth G. Walsh, Michael T.M. Finn, Anna M. Ryden, Lindsey C. McKernan

**Affiliations:** ^1^ Department of Psychology, George Mason University, Fairfax, Virginia, USA.; ^2^ Department of Psychiatry and Behavioral Sciences, Vanderbilt University School of Medicine, Nashville, Tennessee, USA.; ^3^ Department of Physical Medicine and Rehabilitation, Vanderbilt University School of Medicine, Nashville, Tennessee, USA.; ^4^ Betz Congenital Heart Center, Helen DeVos Children’s Hospital of Corewell Health, Grand Rapids, Michigan, USA.; ^5^ College of Human Medicine, Michigan State University, East Lansing, Michigan, USA.

**Keywords:** interstitial cystitis, painful intercourse, psychometrics, self-efficacy

## Abstract

**Background:**

Although female sexual dysfunction (FSD) and low sexual self-efficacy are common in patients with interstitial cystitis/bladder pain syndrome (IC/BPS), existing measures of these constructs do not fully capture unique challenges faced by patients with IC/BPS, such as managing sex-related symptom exacerbations, experiencing rewarding sexual activity, and maintaining intimate relationships. To address the lack of tailored measurement of FSD and sexual self-efficacy in patients with IC/BPS, we aimed to adapt the Painful Intercourse Self-Efficacy Scale—Interstitial Cystitis (PISES-IC) for this population.

**Method:**

To form the PISES-IC, we added three items to the pain self-efficacy subscale of the PISES, each informed directly by qualitative interviews with patients with IC/BPS and literature review of patient-reported sexual experiences in IC/BPS. Utilizing baseline data of 71 female participants involved in a clinical trial for IC/BPS (NCT#04275297), we assessed the validity and reliability of the newly adapted PISES-IC.

**Results:**

Results indicate that the PISES-IC is indeed a valid and reliable measure of sexual self-efficacy in the IC/BPS population and that the items informed by IC/BPS patient experiences (self-efficacy related to pain flares, rewarding sexual activity, and interference with romantic relationships) may be particularly related to FSD in patients with IC/BPS.

**Conclusions:**

The PISES-IC captures aspects of sexual experiences of patients with IC/BPS that are not assessed by other existing measures. The PISES-IC can be utilized in research and clinical settings to inform patient care and to further understand sexual experiences of IC/BPS patients.

## Introduction

Interstitial cystitis/bladder pain syndrome (IC/BPS) is marked by urinary symptoms and pelvic pain, impacting sexual functioning and quality of life.^1,2^ The association between IC/BPS and sexual dysfunction is well established in both the empirical literature^3,4^ and in patients’ descriptions of their disease-related distress and impairment.^5,6^ However, there is no existing measure of female sexual dysfunction (FSD) tailored to patients with IC/BPS, resulting in limited understanding of how FSD presents and impacts well-being within this population.

Despite the pervasive impacts of IC/BPS on sexual functioning, recent reviews of the literature have identified a need to better understand sexual dysfunction in IC/BPS.^4^ Patients with IC/BPS may experience decreased desire, arousal, and orgasm frequency, as well as dyspareunia before and after intercourse.^7,8^ Additionally, it is common for patients to fear that sex may further exacerbate existing bladder symptoms that are already significantly disruptive to their quality of life, and sexual intercourse can serve as a trigger provoking pain flares.^1,9^ Though sexual dysfunction is commonly comorbid with IC/BPS, it often goes undertreated or unaddressed altogether, with fewer than half of IC/BPS treatment studies producing significant change in sexual functioning.^3^ Many health care providers and patients report that sexual health is not adequately addressed in treatment due to deficits in provider education and barriers such as stigma and provider concern about patient discomfort discussing such a sensitive topic.^10–12^

Patients with IC/BPS emphasize the negative impacts of IC/BPS symptoms on their sexual functioning,^5,6^ but the unique experiences of patients with IC/BPS are not adequately assessed in the most commonly used measure of sexual dysfunction. The Female Sexual Functioning Index (FSFI) was developed to assess desire, arousal, lubrication, orgasm, satisfaction, and pain and is frequently utilized to assess sexual dysfunction in IC/BPS.^13,14^ Individuals with IC/BPS report dreading or avoiding sexual intercourse for fear of pain and symptom exacerbation. They also describe the negative impacts of intimacy avoidance on their romantic relationships, recounting discomfort communicating with their partners about their fears of sexual activity, or avoiding or even terminating relationships due to their symptoms.^5,6^ While the FSFI assesses components of sexual function that are indeed relevant to patients with IC/BPS, it fails to address the aspects that patients with IC/BPS describe as being most central to their sexual well-being.^5,6^ Furthermore, the FSFI may not capture the experiences of people that are suffering the most sexual dysfunction; if patients are avoiding sex altogether, then constructs measured in the FSFI such as ability to orgasm and lubrication are rendered less relevant. Thus, it is crucial to consider aspects of sexual dysfunction that are relevant regardless of one’s recent engagement (or lack thereof) in sexual activity.

An important yet understudied aspect of sexual function is sexual self-efficacy or belief in one’s own ability to manage sexual dysfunction and related pain. Although sexual self-efficacy has not been formally assessed within the IC/BPS population, in other populations (such as patients with endometriosis and/or provoked vestibulodynia), it has been shown to positively impact quality of life, mediate the relationship between attachment anxiety and pain intensity, and predict decreased pain intensity, increased sexual satisfaction, and decreased catastrophizing.^15–18^ Importantly, several studies have demonstrated that sexual self-efficacy is a modifiable target that can be significantly reduced with noninvasive interventions such as physical therapy,^19^ group cognitive behavioral therapy,^20^ and even a single-day workshop focused on increasing patient knowledge.^21^ Sexual self-efficacy is a modifiable target with clear impact on quality of life and sexual health; thus, accurate, reliable, and efficient measurement of this construct is vital.

Sexual self-efficacy has been assessed in other populations with chronic pelvic pain utilizing the Painful Intercourse Self-Efficacy Scale^18^ (PISES), but this measure has not been adapted for an IC/BPS population, which features distinct symptom profiles impacting sexual function. The 20-item PISES includes three subscales capturing three aspects of sexual self-efficacy: pain during intercourse, controlling other symptoms, and sexual function. The PISES shows strong reliability,^18^ but a full psychometric evaluation has not been completed. Furthermore, the length of the PISES limits clinical utility. Finally, it may not be appropriate for the IC/BPS population. Patients with IC/BPS report unique challenges impacting their sexual functioning (and likely sexual-self efficacy), such as fear of sexual intercourse leading to pain flares or otherwise impacting their IC/BPS symptoms. Thus, an adapted version of this measure is needed to capture this construct in patients with IC/BPS for both research and clinical purposes.

This study sought to adapt the PISES for women with IC/BPS and to assess psychometric validity of this new scale based on both reviews of the literature and qualitative information gathered from patients with IC/BPS through focus groups.^5^ We hypothesized that our newly adapted measure, the Painful Intercourse Self-Efficacy Scale—Interstitial Cystitis (PISES-IC), would be a valid and reliable measure of sexual self-efficacy in this population.

## Methods

### Study design

This study was a secondary analysis of a registered longitudinal randomized clinical trial (NCT#04275297) conducted at a large regional academic medical center in accordance with the declaration of the World Medical Association and was approved by the institutional review board. For full description of the full trial study methods, see McKernan et al.^22^

Participants were recruited in outpatient clinics, flyers distributed throughout the community, and online via listservs, social media, and ResearchMatch.^23^ In the full study, participants were randomized to receive either eight weekly sessions of an IC/BPS-specific psychosocial self-management program via telemedicine or eight weekly symptom monitoring phone calls. While participants in the original trial completed measures at three time points, only data from baseline were used for the current study. All measures were completed online through REDCap.^24^ This investigation examined a subset of baseline study measures pertaining to sexual functioning.

### Sample

The current study sample included 71 individuals assigned female at birth with IC/BPS. Eligible individuals were English-speaking adults with IC/BPS, as determined through RAND Interstitial Cystitis Epidemiology sensitivity criteria^25^ and participants’ self-report of IC/BPS diagnosis. Exclusion criteria were: (1) comorbid neurological conditions; (2) active cancer treatment; (3) having cancer-related pain; (4) endorsing active major medical issues or reporting having comorbid primary psychotic or major thought disorders; (5) history of psychiatric hospitalization for issues unrelated to suicidal/homicidal ideation or posttraumatic stress; (6) active auditory or visual hallucinations, substance misuse, or acute psychological distress; (7) communication difficulties over the phone; or (8) participants undergoing active individual psychotherapy. These exclusionary factors could either modify study results or affect a person’s ability to reliably complete study procedures.

### Measures

Participants reported their age, race, age of IC/BPS symptom onset, age of IC/BPS diagnosis, sex assigned at birth, identified gender, and other demographic variables (Table 1). Sexual functioning was measured with the FSFI,^14^ a widely used 19-item measure of FSD captured across six subscales: desire, arousal, lubrication, orgasm, satisfaction, and pain. This measure is reliable, shows good discriminant validity in pelvic pain populations,^26^ and has been widely used in IC/BPS.^14^

**Table 1.** Participant Demographics

Variable	*n* (%)	*M* (SD) [min, max]
Age		44.32 (13.54) [21, 70]
Age at diagnosis		34.64 (13.16) [14, 67]
Age symptoms began		28.03 (15.31) [1, 65]
Identified gender		
Woman	70 (98.6%)	
Man	1 (1.4%)	
Sex assigned at birth		
Female	71 (100%)	
Relationship status		
Single, never married	14 (19.7%)	
Married or partnered	44 (62%)	
Divorced	10 (14.1%)	
Separated	1 (1.4%)	
Widowed	1 (1.4%)	
Race/ethnicity		
African American/Black	2 (2.8%)	
Asian/Pacific Islander	2 (2.8%)	
Hispanic/Latinx	1 (1.4%)	
Multiracial	2 (2.8%)	
Native American/American Indian	0 (0%)	
White non-Hispanic	63 (88.7%)	
Prefer not to respond	1 (1.4%)	
Other/Not listed	0 (0%)	
Opioid status		
No	58 (81.7%)	
Yes	12 (16.9%)	

M, mean; SD, standard deviation.

#### Painful Intercourse Self-Efficacy Scale—Interstitial Cystitis

Sexual self-efficacy was assessed through our newly adapted measure, the PISES-IC. The PISES-IC optimized a subscale of the original PISES, tailored to the IC/BPS population.^18^ The original PISES is a 20-item measure assessing three aspects of self-efficacy: a person’s self-efficacy to manage pain during sex, to engage in sexual activity, and for controlling other symptoms such as sexual desire or arousal. Each item is scored from very uncertain (10) to very certain (100), with items summed into total scores and subscales, with lower scores indicating poor self-efficacy. This scale was adapted from the Perceived Self-Efficacy in Arthritis scale.^27^

We opted to utilize one subscale of the original 20-item PISES for brevity both to reduce patient burden and improve clinical utility for IC/BPS. Because bladder/pelvic pain is a key facet of IC/BPS and participants in past qualitative research indicated that managing pain from intercourse was particularly impactful on their quality of life,^5^ we chose to use the pain subscale of the PISES. Notably, the pain subscale of the PISES has demonstrated acceptable reliability across studies.^15,18,19^

More specifically, the optimization of the PISES-IC was informed by a series of qualitative investigations assessing the impact and treatment needs of IC/BPS.^5^ Patient stakeholders acknowledged significant issues with their sex lives, heavily focusing on the consequences that flares and dyspareunia had to romantic relationships. Further questioning revealed that their sexual experiences are largely affected by fear of pain flares, lack of coping strategies to manage symptom flares after sex, and the subsequent inability to experience joy from intimacy due to fear of pain leading to significant relationship tension. A comprehensive literature review found no existing, validated measure of sexual functioning to fully capture this information on issues with painful sex. Thus, in addition to the original five items of the PISES pain subscale, we added the following three items: (1) “How certain are you that you can keep pain from intercourse from leading to IC/BPS symptom increase (a ‘flare’)?” (2) “How certain are you that you can engage in rewarding intimate or sexual activity?” and (3) “How certain are you that you can keep pain from intercourse from interfering with your romantic relationships?”

### Data analytic plan

This investigation intended to describe, validate, and assess the meaningfulness of the newly described scale items developed for IC/BPS. Multiple analyses were performed: We first produced basic descriptives of the PISES-IC in order to assess distribution of scores. Exploratory factor analysis then assessed the measure’s factor structure. In order to assess validity of the PISES-IC, we correlated it with each subscale of the FSFI. To capture reliability, we assessed Cronbach’s alpha for the entire scale and for the scale if each item were not included; we also assessed item-total correlations for each item on the PISES-IC. To examine the additive value of including our three new questions about pain self-efficacy in IC/BPS, we used hierarchical linear regression predicting FSFI, with the sum of the original PISES items and the sum of our three newly added IC/BPS-specific items entered in separate blocks.

## Results

### Sample description

Seventy-one female participants (*m* = 44.32 [standard deviation [SD] =13.54] years) with IC/BPS were eligible for inclusion and completed study measures. See Table 1 for patient demographic characteristics.

### PISES-IC descriptives

Scores on the PISES-IC ranged from a 10 to 100 with a mean of 50.28 (SD = 22.46). Scores were approximately normally distributed, with a mode of 40. Participants who endorsed being sexually active in the past month (n = 46) scored significantly higher on the PISES-IC than those who reported no sexual activity in the past month (n = 15), *t*(59)=3.39, *p* < 0.001.

### Reliability

The PISES-IC showed strong internal reliability (ɑ = 0.91). Table 2 presents item-total correlations as well as reliability statistics if each item were deleted. There was some variation in item-total correlations, all greater than *r* = 0.50.

**Table 2.** Item-Level Reliability Statistics of Painful Intercourse Self-Efficacy Scale—Interstitial Cystitis

Item no.	Item	Item-total correlation	Cronbach’s alpha if item deleted
1	How certain are you that you can decrease your pain from intercourse quite a bit?	0.70	0.90
2	How certain are you that you can continue most of your daily activities?	0.51	0.92
3	How certain are you that you can keep pain from intercourse from interfering with your sleep?	0.73	0.90
4	How certain are you that you can make a small-to-moderate reduction in your pain from intercourse by using methods other than taking extra medicine?	0.76	0.90
5	How certain are you that you can make a large reduction in your pain from intercourse by using methods other than taking extra medication?	0.87	0.89
6	How certain are you that you can keep pain from intercourse from leading to IC/BPS symptom increase (a “flare”)?^a^	0.78	0.90
7	How certain are you that you can engage in rewarding intimate or sexual activity?^a^	0.69	0.91
8	How certain are you that you can keep pain from intercourse from interfering with your romantic relationships?^a^	0.74	0.90

^a^ Newly added item.

IC/BPS, interstitial cystitis/bladder pain syndrome.

### Factor structure

**FIG. 1.**
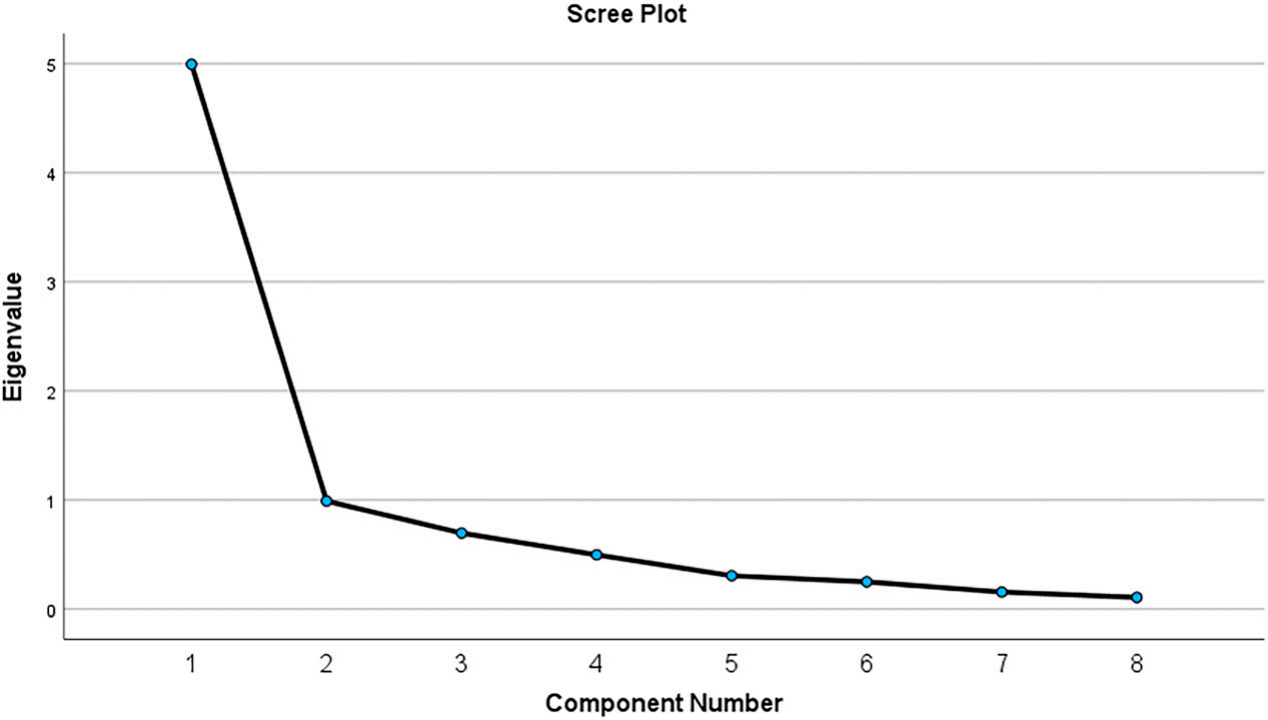
Scree plot of exploratory factor analysis of PISES-IC. PISES-IC, Painful Intercourse Self-Efficacy Scale—Interstitial Cystitis.

Results of an exploratory factor analysis indicated that a unifactor structure fit the data (see Table 3 and Fig. 1). The analysis was a principal components extraction, retaining factors with eigenvalues greater than 1, employing varimax rotation, and excluding cases pairwise.

**Table 3.** Pattern Factor Loadings for Exploratory Factor Analysis of Painful Intercourse Self-Efficacy Scale—Interstitial Cystitis

Item no.	Item	Factor 1
1	How certain are you that you can decrease your pain from intercourse quite a bit?	0.786
2	How certain are you that you can continue most of your daily activities?	0.579
3	How certain are you that you can keep pain from intercourse from interfering with your sleep?	0.796
4	How certain are you that you can make a small-to-moderate reduction in your pain from intercourse by using methods other than taking extra medicine?	0.829
5	How certain are you that you can make a large reduction in your pain from intercourse by using methods other than taking extra medication?	0.919
6	How certain are you that you can keep pain from intercourse from leading to IC/BPS symptom increase (a “flare”)?	0.850
7	How certain are you that you can engage in rewarding intimate or sexual activity?	0.757
8	How certain are you that you can keep pain from intercourse from interfering with your romantic relationships?	0.807

Extraction method: Principle components. Rotation method: Varimax rotation.

### Evidence for convergent/discriminant validity

The PISES-IC showed significant moderate correlations with each subscale of the FSFI (see Table 4).

**Table 4.** Correlations Between Painful Intercourse Self-Efficacy Scale—Interstitial Cystitis and Female Sexual Functioning Index Subscales

Variable	FSFI—Desire	FSFI—Arousal	FSFI—Lubrication	FSFI—Orgasm	FSFI—Satisfaction	FSFI—Pain
PISES-IC	0.367^**^	0.468^**^	0.443^**^	0.453^**^	0.458^**^	0.635^**^
FSFI—Desire	—	0.586^**^	0.480^**^	0.510^**^	0.497^**^	0.420^**^
FSFI—Arousal	—	—	0.807^**^	0.900^**^	0.774^**^	0.647^**^
FSFI—Lubrication	—	—	—	0.793^**^	0.629^**^	0.693^**^
FSFI—Orgasm	—	—	—	—	0.753^**^	0.646^**^
FSFI—Satisfaction	—	—	—	—	—	0.668^**^

^*^ *p* < 0.05.

^**^ *p* < 0.01.

FSFI, Female Sexual Functioning Index; PISES-IC, Painful Intercourse Self-Efficacy Scale-Interstitial Cystitis.

### Exploratory hierarchical regression

As an exploratory analysis, we ran a hierarchical linear regression to determine if the inclusion of the items unique to the PISES-IC explained sexual dysfunction above and beyond the total of the five original PISES-pain items. We entered the sum of the five original PISES items in the first block and the sum of the three novel PISES-IC items in the second block.

The first model was significant, *F*(1, 60) = 21.62, *p* < 0.001, *R*^2^ = 0.27. The original PISES items significantly predicted sexual dysfunction, *b* = .04, β = 0.52, *t* = 4.65, *p* < 0.001. The second model including the addition of the PISES-IC items was significant, *F*(2, 59) *=* 14.09, *p* < 0.001. Including PISES-IC items significantly improved the model, Δ*R*^2^ = 0.06, ΔF(1, 59)= 5.09, *p* = 0.03. With the inclusion of the PISES-IC items, the relationship between the original PISES items and sexual dysfunction was no longer significant, *b* = 0.02, β = 0.23, *t* = 1.43, *p* = 0.16, while the PISES-IC items significantly predicted sexual dysfunction, *b* = 0.05, β = 0.37, *t* = 2.26, *p* = 0.03.

## Discussion

This study adapted a measure of sexual self-efficacy that accurately and reliably captures experiences related to painful intercourse in people with interstitial cystitis. Despite FSD being both common and significantly impacting quality of life in patients with IC/BPS, relatively little research has focused on measuring how sexual dysfunction manifests in IC/BPS and patients’ ability to self-manage issues related to sexual pain. The brief measure we adapted provides an accurate and reliable tool for use in addressing this gap in the research.

The PISES-IC is a novel, valid measure of sexual self-efficacy in IC/BPS and captures aspects of sexual functioning not assessed by the FSFI. The PISES-IC demonstrated convergent validity by positively relating to all subscales of the FSFI. However, the moderate correlations with each subscale suggest the PISES-IC captures unique aspects of sexual dysfunction not reflected by the FSFI alone. The PISES-IC showed the strongest relationship with the pain subscale of the FSFI, indicating that greater pain experienced during intercourse is associated with lower confidence in managing pain. However, correlations between the PISES-IC and the remainder of the FSFI subscales were under 0.50, providing evidence that sexual self-efficacy is distinct from other aspects of sexual function such as desire.

Importantly, low sexual self-efficacy does not equate to low desire for sex. The PISES-IC showed the weakest association with the Desire subscale of the FSFI; this may be a critical facet to capture in an IC/BPS population. While sexual self-efficacy does relate to desire to engage in sex, patients may still *want* sex and yet continue to avoid sex to mitigate symptom exacerbation, a form of compensatory coping.^28^ What’s more, participants who were not sexually active in the past month scored significantly lower on the PISES-IC than those who reported sexual activity in the past month. It is critical to be able to assess sexual function in patients regardless of whether or not they are currently sexually active, but because it is not possible to score the FSFI (except for the Desire subscale) for sexually inactive individuals, many patients who struggle with sexual self-efficacy and sexual dysfunction may be disregarded by the FSFI.

Examining item loadings on the PISES-IC lends insight into other vital aspects of sexual dysfunction that might be missed by existing measures. The results of our exploratory factor analysis indicate that having self-management tools for pain and resources for managing pain flares are particularly important aspects of patients’ sexual self-efficacy. Patients *want* additional tools to manage their symptoms outside of medication.^5^ The majority of patients with IC/BPS experience pain either during or following sex,^1^ and pain flares can be highly variable and debilitating. Additionally, recurrent urinary tract infections are common among patients with IC/BPS^29^ and may both be triggered by sexual activity and trigger or exacerbate IC flares. Patients believe that having management strategies enhances their sense of control over pain experiences, potentially reducing anticipatory distress and avoidance. Patients with IC/BPS are in need of both pharmacological and non-pharmacological tools to manage their pain flares, as patient confidence in keeping pain from intercourse to leading to a pain flare was the second-highest loading item on the factor analysis. Previous research has established the crushing impact of IC/BPS flares, and as a feature unique to IC/BPS, it is critical to address mechanisms perpetuating flares or distress due to flares.

Aspects of sexual self-efficacy identified directly by patients with IC/BPS in focus groups show the strongest relationship to other measures of sexual dysfunction. The total of the three items that were added to the original PISES Pain subscale based on patient report of experiences^5^ predicted sexual dysfunction as captured by the FSFI above and beyond the sum of the original items. Indeed, once the newly added items were included in the model, the relationship between the original items and the FSFI was no longer significant. It appears that managing pain flares, confidence in ability to engage in rewarding sexual activity, and confidence in one’s ability to prevent sexual pain from negatively impacting sexual relationships are of paramount importance in the sexual function of patients with IC/BPS. These findings underscore the importance of utilizing a measure of sexual self-efficacy adapted specifically for an IC/BPS population.

The PISES-IC is an efficient tool to employ in the clinical setting, and focusing on patients’ experience of sexual intercourse is an important clinical target in the service of improving quality of life. Prior research indicates that patients want further communication with their health care providers about their sex lives.^12^ However, the length of other measures of sexual dysfunction such as the original PISES (20 items) and FSFI (19 items) limits their efficiency in a clinical setting. Clinicians could administer the 8-item PISES-IC to patients in order to (1) identify patients who might benefit from targeted intervention to bolster their sexual self-efficacy, (2) initiate a conversation about patients’ sexual self-efficacy and sexual functioning, and (3) tailor both pharmacological and nonpharmacological approaches individually.

### Strengths and limitations

This study targeted measurement of a crucial construct in the understanding of sexual functioning in IC/BPS in a way that has not previously been accomplished. The novel items added to the PISES were directly informed by the voices of those that live with IC/BPS, and analyses confirmed that these items are particularly relevant to sexual functioning for this population.

Although informed by qualitative interviews regarding IC symptoms and their impact, this measure is primarily pain-focused. Individuals with IC can also experience interference to sexual functioning due to urinary urgency^30^ and co-occurring vulvodynia.^30,31^ These associated symptoms may be unmeasured factors that also influence a person’s sexual self-efficacy for some patients.

The study sample was diagnostically homogenous, with assessment of only patients currently meeting criteria for active interstitial cystitis, which may miss important aspects of those who have well-managed symptoms of IC/BPS. Furthermore, this study was limited to participants assigned female at birth and did not measure potentially painful features of intercourse for those with male genitalia. In addition, further consideration of sexual orientation and sexual preferences (e.g., desire to have penetrative vaginal sex or not) would add patient-oriented nuance to barriers to and goals of satisfying sexual experiences. Finally, the most rigorous psychometric analyses of novel measures examine convergent and discriminant validity with the comparison of many extant measures. This area is so historically understudied that few relevant measures exist, and we were only able to utilize one measure of sexual dysfunction to provide evidence of validity.

### Future directions

Further research is needed to better understand how sexual self-efficacy presents within patients with IC/BPS and how it impacts symptom profiles and treatment outcomes. The PISES-IC could be utilized in future studies in order to better understand what leads to lowered self-efficacy, what are the consequences of low sexual self-efficacy, and what interventions can be tailored to increase sexual self-efficacy. In particular, further studies should investigate how sexual self-efficacy contributes to outcomes in patients with IC/BPS above and beyond other aspects of sexual functioning.

While we developed this measure specifically from qualitative research with patients with IC/BPS, only one of the items explicitly references IC/BPS, item six: “How certain are you that you can keep pain from intercourse from leading to IC/BPS symptom increase (a ‘flare’)?”. This item could be potentially adapted in future studies outside of IC/BPS literature by simply removing “IC/BPS” from the item text for initial assessments of reliability and validity. This is potentially a hidden strength of this measure, in that by tailoring closely to the expert IC/BPS experience, it may have captured well the general experience of sexual pain self-efficacy relevant to other contexts as well.

## Conclusion

This research study extends the extant literature by providing a measure of sexual self-efficacy tailored for an IC/BPS population. By presenting the optimized, valid, and reliable PISES-IC, the authors hope to increase the breadth of FSD assessment in patients with IC/BPS, add depth to our understanding of patient’s experiences, and improve intervention for the treatment of FSD in IC/BPS.

## Abbreviations Used

FSD: Female Sexual Dysfunction
FSFI: Female Sexual Functioning Index
IC/BPS: Interstitial Cystitis/Bladder Pain Syndrome
PISES-IC: Painful Intercourse Self-Efficacy Scale - Interstitial Cystitis
